# DNA-PAINT Imaging Accelerated by Machine Learning

**DOI:** 10.3389/fchem.2022.864701

**Published:** 2022-05-10

**Authors:** Min Zhu, Luhao Zhang, Luhong Jin, Jincheng Chen, Yongdeng Zhang, Yingke Xu

**Affiliations:** ^1^ Key Laboratory of Biomedical Engineering of Ministry of Education, State Key Laboratory of Modern Optical Instrumentation, Zhejiang Provincial Key Laboratory of Cardio-Cerebral Vascular Detection Technology and Medicinal Effectiveness Appraisal, Department of Biomedical Engineering, Zhejiang University, Hangzhou, China; ^2^ Alibaba-Zhejiang University Joint Research Center of Future Digital Healthcare, Hangzhou, China; ^3^ School of Life Sciences, Westlake University, Hangzhou, China; ^4^ Binjiang Institute of Zhejiang University, Hangzhou, China; ^5^ Department of Endocrinology, The Affiliated Sir Run Run Shaw Hospital, Zhejiang University School of Medicine, Hangzhou, China

**Keywords:** DNA-PAINT, machine learning, super-resolution imaging, U-Net, single-molecule localization microscopy

## Abstract

DNA point accumulation in nanoscale topography (DNA-PAINT) is an easy-to-implement approach for localization-based super-resolution imaging. Conventional DNA-PAINT imaging typically requires tens of thousands of frames of raw data to reconstruct one super-resolution image, which prevents its potential application for live imaging. Here, we introduce a new DNA-PAINT labeling method that allows for imaging of microtubules with both DNA-PAINT and widefield illumination. We develop a U-Net-based neural network, namely, U-PAINT to accelerate DNA-PAINT imaging from a widefield fluorescent image and a sparse single-molecule localization image. Compared with the conventional method, U-PAINT only requires one-tenth of the original raw data, which permits fast imaging and reconstruction of super-resolution microtubules and can be adopted to analyze other SMLM datasets. We anticipate that this machine learning method enables faster and even live-cell DNA-PAINT imaging in the future.

## Introduction

Super-resolution microscopy allows for optical imaging beyond Abbe’s diffraction limit, enabling the visualization of subcellular structures up to the molecular scale. Super-resolution approaches can be roughly divided into two types, illumination pattern-based microscopy and single-molecule localization microscopy (SMLM) (Schnitzbauer et al., 2017). DNA point accumulation in nanoscale topography (DNA-PAINT) is a promising SMLM method. It requires transient binding of short dye-labeled oligonucleotides to their complementary target strands, which creates the necessary “blinking” to enable stochastic super-resolution imaging of nanoscale structures (Guo et al., 2019; Huang et al., 2020). Different from other SMLM (e.g. PALM and STORM) techniques, the pool of fluorophores for DNA-PAINT could be continuously replenished from the imaging buffer, thus eliminating the concerns over photo-budget. Previous work has shown that DNA-PAINT could achieve a 5 nm localization accuracy with bright fluorescence and longer imaging time (Liu et al., 2019). Therefore, conventional DNA-PAINT requires a large scale of raw data, typically more than 10,000 images, to reconstruct one super-resolution image (Clowsley et al., 2020), which prevents it from being applied to subcellular structure imaging in live cells (Schlichthaerle et al., 2017; Brockman et al., 2020).

Machine learning is a data-oriented method, which can perform complicated tasks by employing artificial neural networks. Machine learning has been applied to increase magnification and resolution in fluorescence microscopy (Wang et al., 2019). A method called ANNA-PALM was demonstrated to only use 1% of raw data to reconstruct one super-resolution image by applying machine learning methods (Ouyang et al., 2018). Localization tasks can be accelerated, and the accuracy can be improved by convolutional neural network (CNN) (Nehme et al., 2018; Cardoen et al., 2020), which is the most commonly used artificial neural network. Machine learning is a potential approach to accelerate DNA-PAINT imaging.

To accelerate DNA-PAINT imaging, we developed U-Net-assisted DNA-PAINT (U-PAINT) based on CNN. We have achieved fast super-resolution imaging of microtubule by applying U-PAINT. This strategy uses one-tenth of frames and independent localizations to reconstruct a super-resolution image without trading off spatial resolution.

## Materials and Methods

### Sample Preparation

COS-7 cells and HeLa cells were cultured in high glucose Dulbecco’s modified Eagle’s medium (DMEM) (Cytiva, SH30243.01B), supplemented with 10% fetal bovine serum (HyClone, SV30087) and 1% penicillin–streptomycin (Beyotime, C0222) at 37°C in a humidified 5% CO_2_ incubator. Cells were grown on a 35-mm glass-bottom dish (Cellvis, D35-20-1-N) for immunostaining experiments.

The detailed procedures for immunofluorescence cell staining were as follows. The cells were first incubated in a pre-fixation buffer (0.4% glutaraldehyde and 0.25% Triton X-100 in PBS) for 90 s at 37°C and then fixed with a fixation buffer (3% glutaraldehyde and 0.25% Triton X-100 in PBS) at room temperature. Fixatives were quenched with newly dissolved 1 mg/ml NaBH_4_ in PBS for 30 min. After washing three times with PBS, the cells were incubated in a blocking buffer (5% BSA and 0.25% Triton X-100 in PBS) for 2 h, rinsed three times, and then incubated with a mouse monoclonal antibody to beta-tubulin (Abcam, ab231082) at a concentration of 1:500 overnight at 4°C. The cells were then incubated with a biotin-labeled goat anti-mouse IgG secondary antibody (Abcam, ab6788) at a concentration of 1:500 for 2 h at room temperature and subsequently incubated with Alexa Fluor 488 conjugated streptavidin (Invitrogen, S32354) at a concentration of 1:1,000 for 15 min at room temperature. After washing three times with PBS, the cells were incubated with biotin-conjugated docking strands (biotin-TTATACATCTATACATCTA) at a concentration of 1 μM for 15 min at room temperature protected from light. Finally, the cells were preserved for imaging in PBS at 4°C.

### DNA-PAINT Imaging

The imaging buffer consists of 0.5 nM Cy3B-conjugated imager strands (TAGATGTAT-Cy3B), 50 mM MgCl_2_, and 50 mM NaCl. An Olympus IX83 microscope was used and set to a TIRF mode with a penetration depth of 200 nm. For widefield imaging, 488-nm laser (5 mW) was used for illumination. Widefield images were acquired under the control of Cellsense software with an exposure time of 100 ms. For DNA-PAINT imaging of the same region, 561-nm laser (100 mW) was used for illumination. A total of 30,000 TIRFM images were acquired with an exposure time of 100 ms.

### DNA-PAINT Image Reconstruction

DNA-PAINT images were reconstructed with Picasso software developed by the Jungmann Lab (Schnitzbauer et al., 2017). Minimal net gradient was set manually to avoid background fluorescence from being localized. After localization, the maximum likelihood estimation was applied for fitting and then fitted localizations with an ellipticity greater than 0.6 were removed. The rest data were rendered into 16 time-scaled 8-bit png files whose pixel size was 8 × 8 nm after a redundant cross-correlation drift correction (RCC). To obtain 16-bit images as the output of reconstruction, R package EBImage (Pau et al., 2010) was used. Reconstructions with 1,000 and 3,000 raw images were performed using R. The R script can be found in the GitHub repository (https://github.com/ccchin999/PAINT-learning).

### Real Microtubule Dataset Preparation

Reconstructed DNA-PAINT images from 30,000 raw frames (ground truth), 1,000 frames, or 3,000 frames (input) were cut by 256 × 256 pixel grids. The cropped images were filtered according to a comparison of mean intensity values of themselves and the whole image. Those with low mean values were excluded to guarantee image quality. And then, widefield images were cut and selected accordingly. We obtained about 1,300 sets of cropped images for data training and an additional 36 ones as the testing dataset. Each set consists of widefield images, reconstructed DNA-PAINT images from 1,000, 3,000, and 30,000 raw images. Reconstructed DNA-PAINT images from 30,000 raw images were treated as the ground truth. Those images were normalized to the maximum intensity of the corresponding uncut images.

PALM image data were downloaded from the ANNA-PALM GitHub repository (Ouyang et al., 2018). PALM data of 60,000 frames was rendered to an image with the same resolution of DNA-PAINT images. The image was then cut by 256 × 256 pixel grids. PALM data of randomly selected 6,000 frames was processed as stated above. The corresponding widefield image was scaled to the same size and cut by 256 × 256 pixel grids. The images with a low mean intensity were removed. We chose 90 pairs of PALM images as testing datasets.

### Simulated Microtubule Datasets

The microtubular structures were stimulated using the random-walk simulation as previously published (Weigert et al., 2018). In the first frame, approximately 10 starting points of trajectories were selected randomly on the boundary of the image. In each frame, microtubules moved a fixed length (about a half-pixel to make trajectories continuous and smooth) toward the center, with the displacement between the nearby positions according to the normal distribution. We simulated 500 frames for one set of images to make sure most microtubule trajectories were across the entire image, which resembles the real data. For ground truth, all these frames were overlaid to one image, which was rescaled, blurred with a Gaussian kernel with a standard deviation of 1.25 pixels, and then cropped into 256-pixel-width squares. For widefield images, 256-pixel-width ground-truth images were blurred with a Gaussian kernel with a standard deviation of 20 pixels, resized to 32-pixel-length squares, and scaled to the same size as original ground-truth images. For sparse localization images, 10% frames were randomly selected and overlaid to one image, rescaled, Gaussian-blurred, and cropped, as stated above. The R script for microtubule simulation can be found at the GitHub repository (https://github.com/ccchin999/PAINT-learning).

### Model Training

We adopted the *Python* package U-Net as previously reported (Jin et al., 2020) and with similar computation platform (Intel Core i9-10900KF CPU and NVIDIA GeForce RTX 3080 GPU). We trained three different models, U-PAINT (3,000), U-PAINT (WF), and U-PAINT (WF+3,000). They share similar network architectures, with the only difference being input channel numbers. The U-PAINT (3,000) model was trained from reconstructed DNA-PAINT images with sparse localization (3,000 raw images). The U-PAINT (WF) model was trained with widefield images, whereas the U-PAINT (WF+3,000) was trained with both sparse localization images and the corresponding widefield image. The reconstructed DNA-PAINT images with 30,000 raw images were used as the ground truth. Each model was trained for 2,000 epochs, which cost approximately 10–15 h. Additionally, we trained U-PAINT (3,000) and U-PAINT (WF+3,000) for 2,000 more epochs with simulated microtubule data and 500 epochs using real data. The additional training spent about 40 h.

### Model Performance Quantification

The peak signal-to-noise ratio (PSNR), root-mean-square error (RMSE), and structural similarity image measurement (SSIM) were used to evaluate the performance of trained models. All values come from the differences between the output (OP) and ground truth (GT) of the testing datasets. The intensity of images was mapped to the interval [0, 255]. PSNR, RMSE, and SSIM were calculated using the following functions, where cov(GT,OP) is referred to as the covariance of GT and OP, sd function is the represented standard deviation, and $c_{1},c_{2}$ are the small positive constants.

$$\begin{array}{ccc} \mathrm{RMSE} & = & \sqrt{\mathrm{mean}\left({\left(\text{G}\text{T}-\mathrm{OP}\right)}^{2}\right)}\\ \mathrm{PSNR} & = & \{\begin{array}{cc} 100 & ,\mathrm{when}\mathrm{RMSE}=0\\ 20\mathrm{lg}\left(\frac{255}{\mathrm{RMSE}}\right) & ,\mathrm{when}\mathrm{RMSE}>0 \end{array}\\ \mathrm{SSIM} & = & \frac{\left[2\mathrm{mean}\left(\text{G}\text{T}\right)\mathrm{mean}\left(\mathrm{OP}\right)+c_{1}\right]\left[\mathrm{cov}\left(\text{G}\text{T},\mathrm{OP}\right)+c_{2}\right]}{\left[\mathrm{mean}{\left(\text{G}\text{T}\right)}^{2}+\mathrm{mean}{\left(\mathrm{OP}\right)}^{2}+c_{1}\right]\left[\mathrm{sd}{\left(\text{G}\text{T}\right)}^{2}+\mathrm{sd}{\left(\mathrm{OP}\right)}^{2}+c_{2}\right]} \end{array}$$

## Results and Discussion

### Improved DNA-PAINT Labeling Method

**FIGURE 1:**
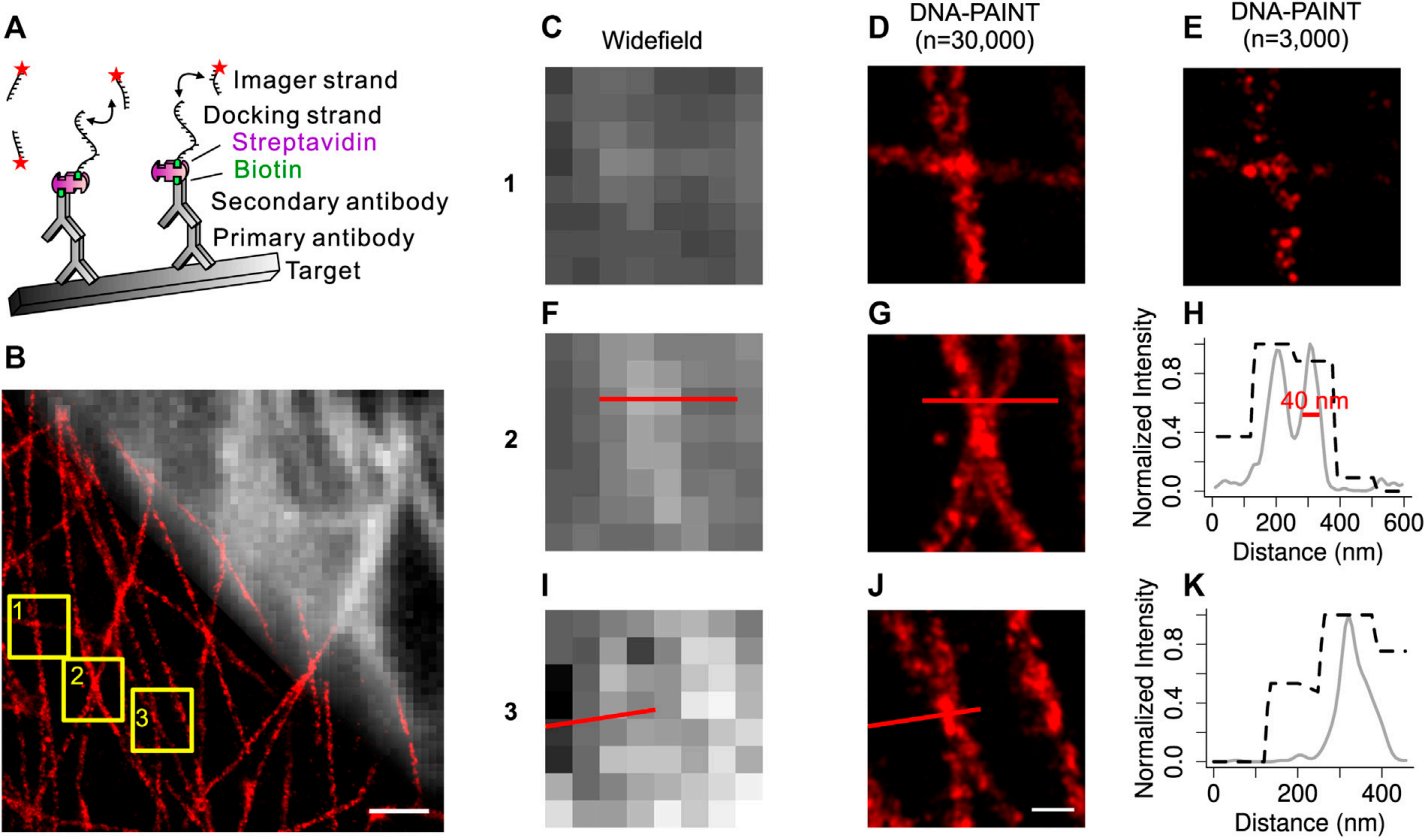
Development of the improved DNA-PAINT labeling method. **(A)** Overview of the improved DNA-PAINT system. Labeling strategy for DNA-PAINT using antibodies, fluorescent dye-conjugated streptavidin, docking strands, and complementary imager strands. **(B)** Overlay of a widefield microtubule image (top right) with its DNA-PAINT reconstruction image (bottom left). **(C) (F) (I)** Widefield images of the boxed regions 1, 2, 3 from **(B) (D) (G) (J)** DNA-PAINT reconstructions (*n* = 30,000) of the same boxed regions 1, 2, 3 from **(B) (E)** Sparse localization image (*n* = 3,000) of **(D) (H)** Normalized intensity plot of regions from F, **(G) (K)** Normalized intensity plot of regions from I, **(J)**. Black dashed curves from widefield images; gray solid curves from DNA-PAINT reconstructions (n = 30,000). Scale bar: 1,000 nm **(B)**, 200 nm **(J)**. Machine learning approach for the DNA-PAINT image reconstruction.

To acquire super-resolution DNA-PAINT images together with widefield images of the same endogenous structures, we developed an improved DNA-PAINT labeling method. This DNA-PAINT system, as illustrated in Figure 1A, uses immunostaining approaches with an Alexa Fluor 488 conjugated streptavidin- and biotin-modified docking strand to target a subcellular structure of interest. The imager strand conjugated to the Cy3B dye can diffuse freely in the imaging buffer. Owing to their complementary sequence, blinking occurs during the transient binding events of imager strands and docking strands. All materials mentioned above have been optimized by commercial companies, and immunostaining is easy to achieve.

With this improved DNA-PAINT system, we carried out widefield and DNA-PAINT imaging of the endogenous microtubules in COS-7 cells (Figure 1B). Comparison of the widefield images and DNA-PAINT images revealed that DNA-PAINT images were consistent with widefield images but with much better spatial resolution. Zoom-in views (Figures 1C,D,F,G,I,J) of three areas confirmed the high resolution of DNA-PAINT images. Quantification of the reconstructed DNA-PAINT image demonstrated that a spatial resolution of 40 nm could be achieved on microtubules (Figures 1H,K). Thus, by using this improved DNA-PAINT system, we could simultaneously obtain the diffraction-limited widefield images and super-resolution DNA-PAINT images of the endogenous microtubules in cells.

**FIGURE 2:**
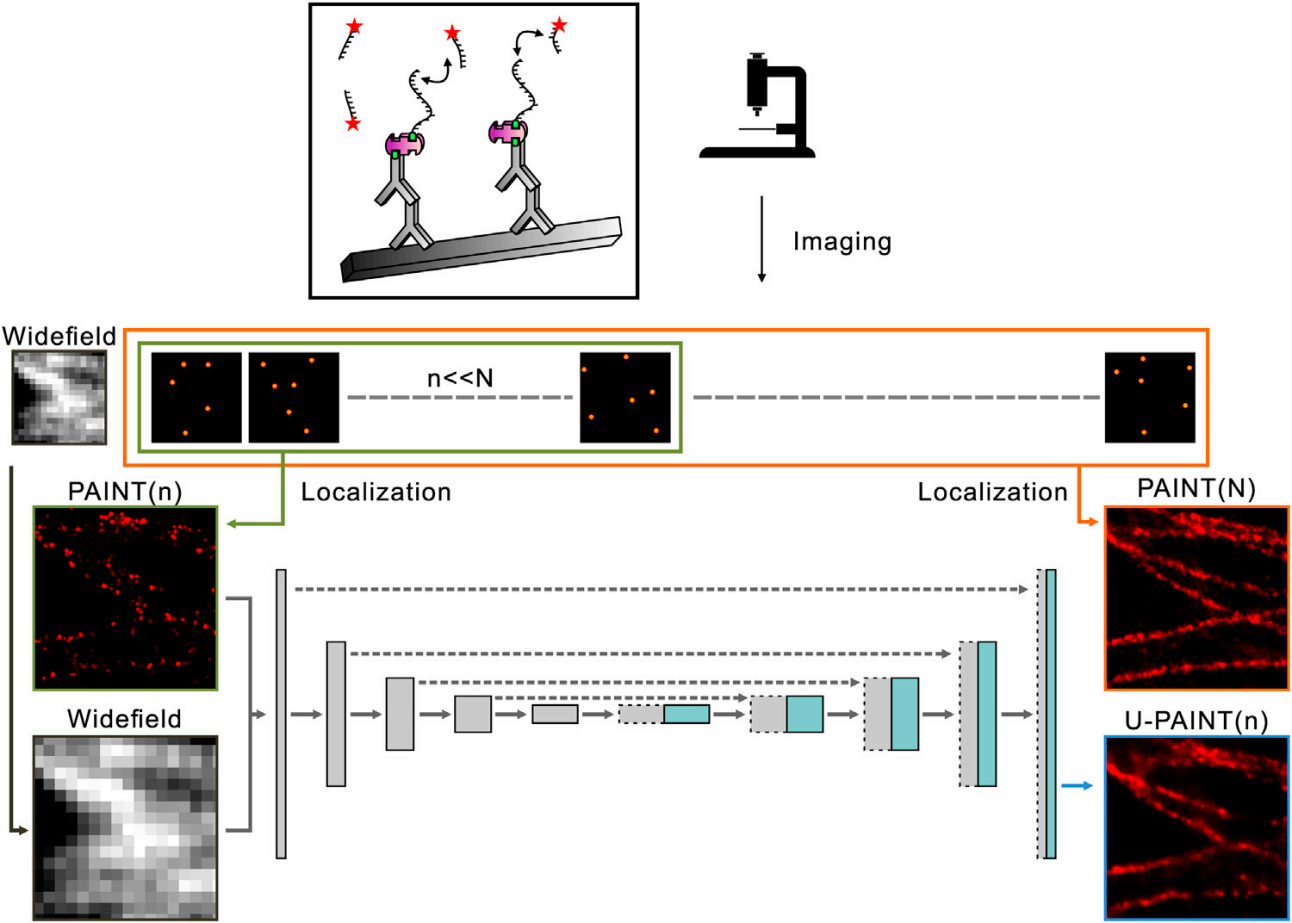
Overview of U-PAINT. Training images are localized and rendered from long sequences of single-molecule image frames or widefield images. Sparse localization images from the first n frames and widefield images are set as inputs of U-PAINT, while dense localization images of N frames (N >> *n*) are outputs. Simulated microtubule data enhance U-PAINT training.

Originally designed for biomedical segmentation, U-Net has proven to be a useful machine learning network architecture and is widely applied to image restoration, classification, and quantification (Ronneberger et al., 2015; Falk et al., 2019; Byra et al., 2020; Clowsley et al., 2020; Yan et al., 2022). To reconstruct a super-resolution image with the resolution similar to a standard DNA-PAINT image but with a much smaller number of blinking points (less raw data), the machine learning method U-PAINT derived from U-Net is developed. U-PAINT contains a total of 10 layers, including four downsampling convolutional layers, four upsampling convolutional layers, an input layer, and an output layer. A slightly more than 31 million parameters are modified through backpropagation algorithms and stochastic gradient descent. As shown in Figure 2, super-resolution images of microtubules (N frames) are obtained by the conventional DNA-PAINT imaging and are processed with Picasso software (Figures 1D,G,J). The widefield images of the same structure can be acquired through imaging the dye Alexa Fluor 488 labeled on streptavidin. Sparse DNA-PAINT images are yielded by using a much smaller number of DNA-PAINT frames (n frames, *n* << N) from the same localization (Figure 1E). Once trained, U-PAINT can be applied to new sparse DNA-PAINT images obtained from image sequences of another microtubule sample with only a few frames in a much shorter time, which can contribute to the reconstruction of high-quality super-resolution images, with or without widefield images.

**FIGURE 3:**
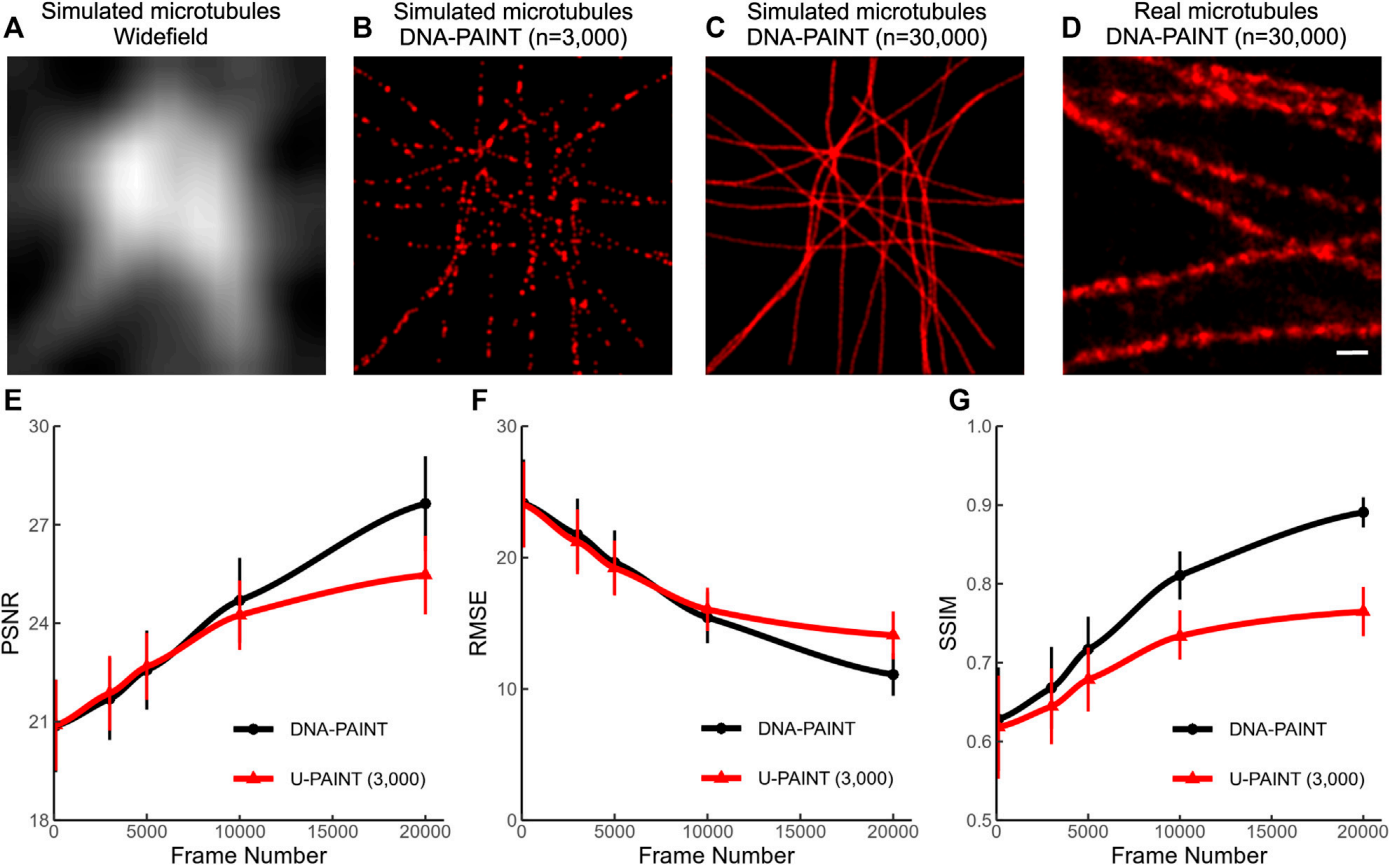
Simulation of microtubules and their reconstruction quality. **(A)** Widefield image of simulated microtubules. **(B)** Sparse localized DNA-PAINT image (*n* = 3,000) of simulated microtubules. **(C)** Dense localized DNA-PAINT image (*n* = 30,000) of simulated microtubules. **(D)** Dense localized DNA-PAINT image (*n* = 30,000) of real microtubules. **(E)** PSNR of U-PAINT (3,000) reconstruction results and DNA-PAINT results with different frame numbers. **(F)** RMSE of U-PAINT (3,000) reconstruction results and DNA-PAINT results with different frame numbers. **(G)** SSIM of U-PAINT (3,000) reconstruction results and DNA-PAINT results with different frame numbers. PSNR, RMSE, and SSIM are measured compared with results and ground truth. Red lines with triangle points: performance of U-PAINT (3,000); black lines with circle points: performance of conventional DNA-PAINT. Dots are averages from 36 simulations; error bars show s.d. Scale bar: 200 nm. U-PAINT reconstruction of endogenous microtubules.

We simulated more than 3,000 artificial microtubule images to satisfy U-PAINT’s demand of a larger training dataset. A random walk-based simulation example is listed in Figure 3C. The widefield image (Figure 3A) was rendered by blurring with a Gaussian kernel of a 20-pixel standard deviation. The sparse localization image (Figure 3B) was generated with only 1 in 10 of the randomly chosen simulation localizations. Those artificial microtubules are more controllable in both continuity and density. However, microtubules are hollow cylinders with ∼26 nm diameter, which is not considered in our simulation. As a result, our simulated microtubules are thinner than real microtubules (Figure 3D). This noticeable difference actually affects our U-PAINT models. After training with artificial microtubule datasets, our models could not restore real microtubules correctly. Thus, we retrained the model for 500 epochs with real microtubule training datasets.

We tested our U-PAINT (3,000) model on simulation data with different frame numbers from 100 to 20,000 (Figures 3E–G). The parameters PSNR, RMSE, and SSIM are quantitatively measured. Higher PSNR implies stronger signals and lesser noise being identified as part of the microtubules. Lower RMSE suggests more precise reconstruction. SSIM lies between 0 and 1 and reaches 1 when reconstruction images are the same as ground truth. The performance of U-PAINT is greatly enhanced with an increased frame number. However, when the frame number is larger than 5,000, U-PAINT (3,000) becomes unable to increase image quality for DNA-PAINT.

**FIGURE 4:**
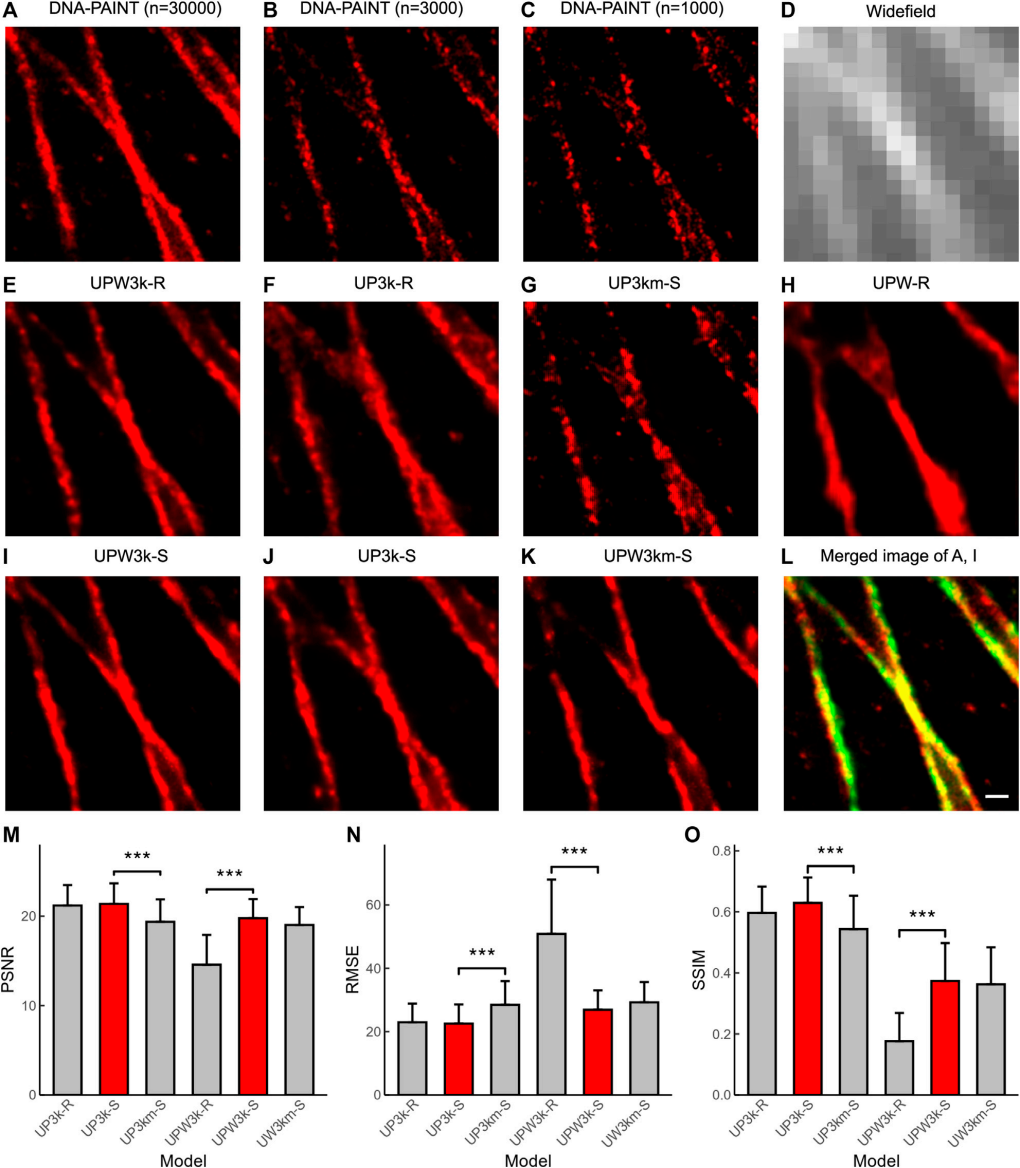
U-PAINT reconstruction of microtubules. **(A)** DNA-PAINT reconstruction images with n = 30,000 raw data. **(B)** DNA-PAINT reconstruction image with n = 3,000 raw data. **(C)** DNA-PAINT reconstruction image with n = 1,000 raw data. **(D)** Widefield image. **(E)** Real data-based U-PAINT (WF+3,000) reconstruction image with B and D as inputs. **(F)** Real data-based U-PAINT (3,000) reconstruction image with B as input. **(G)** Real data-based U-PAINT (1,000) reconstruction image with C as input. **(H)** Real data-based U-PAINT reconstruction image with **(D) (I)** Simulated and real data-based U-PAINT (WF+3,000) reconstruction image with B and D as inputs. **(J)** Simulated and real data-based U-PAINT (3,000) reconstruction image with B as input. **(K)** Simulated and real data-based U-PAINT (WF+3,000) reconstruction image with C and D as inputs. **(L)** Merged image of A (red) and I (green). **(M)** PSNR of U-PAINT models. **(N)** RMSE of U-PAINT models. **(O)** SSIM of U-PAINT models. Red bars refer to better U-PAINT models; bars are average values from testing dataset; error bars show s.d.; UP3k-R: U-PAINT (3,000) model trained with only real data; UP3k-S: U-PAINT (3,000) model trained with real and simulated data; UP3km-S: U-PAINT (3,000) model trained with real and simulated data, and the input was a sparse DNA-PAINT image localized from 1,000 frames of raw data; UPW3k-R: U-PAINT (WF+3,000) model trained with only real data; UPW3k-S: U-PAINT (WF+3,000) model trained with real and simulated data; UPW3km-S: U-PAINT (WF+3,000) model trained with real and simulated data, and the inputs are a widefield image and a sparse DNA-PAINT image localized from 1,000 frames of raw data. *** represents *p* < 0.001 as tested by two independent sample *t*-test; scale bar: 200 nm. Comparison between ANNA-PALM and U-PAINTs.

We tested U-PAINT on immunostained microtubules. DNA-PAINT images with the corresponding widefield images were obtained during a 50-min-long imaging (N = 30,000; Δt = 100-ms exposure time) (Figures 4A,D). The sparse DNA-PAINT images were obtained from only 5- or 2-min imaging (*n* = 3,000 or 1,000) (Figures 4B,C). Although microtubule filaments can already be seen in sparse DNA-PAINT images, structural details below the diffraction limit are hard to discern, making it difficult to identify features such as filament crossings (Figures 4B,C). We first tried to reconstruct super-resolution images from widefield images. Our results show that a few structures are restored (Figure 4H; UPW-R refers to U-PAINT (WF) trained with only real data), which means that precise restoration requires super-resolution localization images. Then, we attempted to restore images from sparsely localized images. After training with ∼1,300 sparse-and-dense image pairs of real microtubules, U-PAINT completed the detailed structures of input sparse DNA-PAINT images (Figure 4F; UP3k-R represents U-PAINT (3,000) model trained with only real data). However, the result is not perfect enough as artifacts cover signals to some extent and reconstructed microtubules are fragmented.

We further tested whether adding matched widefield images with DNA-PAINT images could promote reconstruction quality. Our results show that adding widefield images brings more continuity and instability. In some cases, widefield images make restored structures more precise and continuous (Figure 4E; UPW3k-R represents U-PAINT (WF+3,000) model trained with only real data), while more severe artifacts were induced otherwise. We continued testing whether the addition of simulated data in the training period could improve reconstruction quality. For U-PAINT (WF+3,000), instability from widefield images is inhibited and progress of continuity can be noticed (UPW3k-R and UPW3k-S in Figures 4M–O; UPW3k-R refers to the U-PAINT (WF+3,000) model trained with only real data and UPW3k-S refers to the U-PAINT (WF+3,000) model trained with real and simulated data). Most structures are restored by U-PAINT (WF+3,000) trained with both real and artificial microtubule data (Figures 4I,L). In addition, we also noticed a slight enhancement for the performance of U-PAINT (3,000) when trained with both real and simulated data (Figures 4F,J; UP3k-S refers to U-PAINT (3,000) trained with real and simulated data).

Then, we tested whether the reconstruction quality could remain for U-PAINT after decreasing the number of input sparse-localized DNA-PAINT frames (n) to 1,000, where the image acquisition time was cut down to only 100 s. Although microtubule filaments appear completely discrete, most structural details below the optical diffraction limit are restored (Figures 4G,K; UP3km-S represents the U-PAINT (3,000) model trained with simulated data and real data and inputs are a sparse DNA-PAINT image localized from 1,000 frames of raw data; UPW3km-S represents the U-PAINT (WF+3,000) model trained with simulated data and real data and inputs are a widefield image and a sparse DNA-PAINT image localized from 1,000 frames of raw data). For U-PAINT (WF+3,000), only a slight decrease in performance is noticed (UPW3k-S and UPW3km-S of Figures 4M–O). However, the performance of U-PAINT (3,000) is reduced significantly (UP3k-S and UP3km-S of Figures 4M–O).

Using testing datasets as input, we obtained 36 output images. The quantitative values of PSNR, RMSE, and SSIM between output and ground truth were calculated and plotted (Figures 4M–O). By the addition of the simulated microtubule data, the U-PAINT (WF+3,000) model is improved, while the U-PAINT (3,000) model has no significant enhancement. When we reduce the input frame number to 1,000, the output quality of U-PAINT (WF+3,000) remains at a relatively high level, while the quality of U-PAINT (3,000) decreases remarkably. Collectively, these results demonstrate the advantage of using both widefield and super-resolution DNA-PAINT images and the importance of including both real data and simulated data in model training.

**FIGURE 5:**
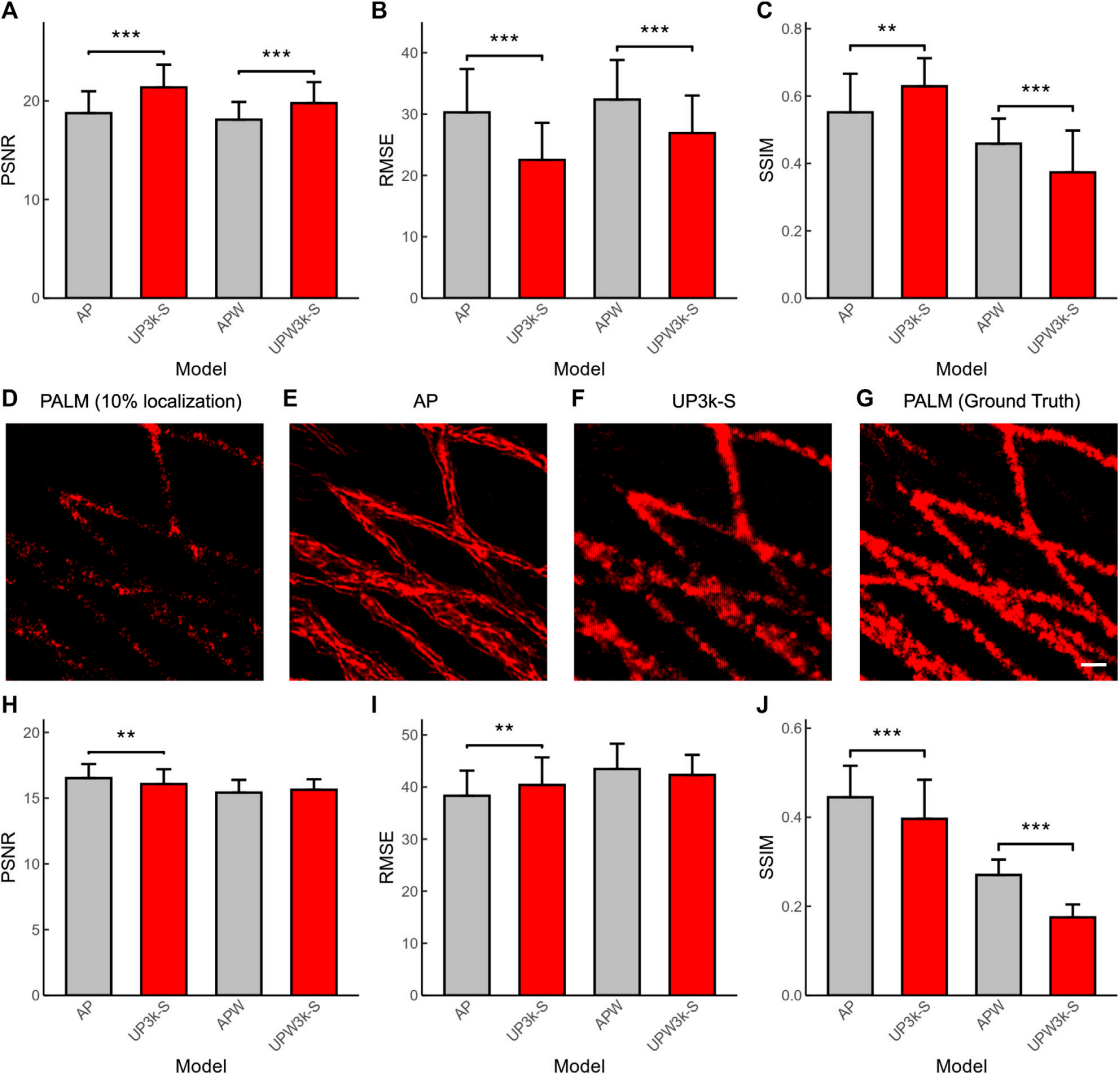
Quantification of the performance of U-PAINT models and ANNA-PALM (AP). The performance of U-PAINT models and AP in DNA-PAINT reconstruction as analyzed by PSNR **(A)**, RMSE **(B)**, and SSIM **(C)**. **(D)** A representative image of sparse-localized PALM as reconstructed by the traditional method. The accelerated reconstruction images by ANNA-PALM **(E)** and U-PAINT **(F)** and the ground-truth image **(G)**. The performance of U-PAINT models and AP in PALM reconstruction as analyzed by PSNR **(H)**, RMSE **(I),** and SSIM **(J)**. Red bars refer to U-PAINT models; bars are average values; error bars show s.d.; ** represents *p* < 0.01 and *** represents *p* < 0.001, as tested by two independent sample *t*-test. AP: ANNA-PALM pre-trained model with a sparse localization image as input; UP3k-S: U-PAINT (3,000) model trained with real and simulated data; APW: ANNA-PALM pre-trained model with a sparse localization image and a widefield image as inputs; UPW3k-S: U-PAINT (WF+3,000) model trained with real and simulated data. Scale bar: 200 nm.

PALM is another SMLM that shares the same reconstruction algorithm with DNA-PAINT. Here, we applied the deep-learning based ANNA-PALM models (AP) (Ouyang et al., 2018) and compared its performance with our established U-PAINT models. The results show that U-PAINT has higher restoration quality (AP and UP3k-S in Figures 5A,B; AP represents ANNA-PALM). Although ANNA-PALM brings better precision of output image quality when both widefield images and sparsely localized images are used as inputs (APW and UPW3k-S in Figure 5C; APW refers to ANNA-PALM with an extra widefield image as input), it restores fewer signals (APW and UPW3k-S in Figure 5A). Thus, we conclude that U-PAINT is more suitable for DNA-PAINT imaging acceleration.

Similarly, we also tested whether our developed U-PAINT models can be used to accelerate PALM imaging (Figures 5D–G). The original PALM images were from the ANNA-PALM GitHub repository (Ouyang et al., 2018). Our result shows that U-PAINT without inputting widefield images is able to accelerate PALM imaging, although its performance quantification indicators are not as excellent as ANNA-PALM (Figures 5H,I,J). However, the restored image of ANNA-PALM shows abnormal artifacts (Figure 5E), which makes the ANNA-PALM reconstruction output unauthentic. Taken together, our U-PAINT model is practical to accelerate SMLM other than DNA-PAINT.

## Conclusion

We introduced a new DNA-PAINT labeling method that allows for imaging of cellular structures with both DNA-PAINT and widefield illumination. We proposed machine learning-based U-PAINT model that manages to reduce the demanded number of raw images for the DNA-PAINT reconstruction of microtubules to less than 10% of the conventional method but still achieving comparable spatial resolution. By co-training with simulated microtubule datasets, we showed that the performance of the U-PAINT model can be further elevated. In addition, our method can be easily transferred to process other types of SMLM, such as PALM, and enables the acceleration of SMLM imaging. Therefore, we anticipate that live-cell DNA-PAINT imaging can be potentially realized for some specific subcellular structures in the future.

## Data Availability

The raw data supporting the conclusion of this article will be made available by the authors without undue reservation.

## References

[B1] Byra M. Jarosik P. Szubert A. Galperin M. Ojeda-Fournier H. Olson L. (2020). Breast Mass Segmentation in Ultrasound with Selective Kernel U-Net Convolutional Neural Network. Biomed. Signal Process. Control. 61, 102027. 10.1016/j.bspc.2020.102027 PubMed Abstract | 10.1016/j.bspc.2020.102027 | Google Scholar 34703489 PMC8545275

[B2] Cardoen B. Yedder H. B. Sharma A. Chou K. C. Nabi I. R. Hamarneh G. (2020). ERGO: Efficient Recurrent Graph Optimized Emitter Density Estimation in Single Molecule Localization Microscopy. IEEE Trans. Med. Imaging 39, 1942–1956. 10.1109/TMI.2019.2962361 PubMed Abstract | 10.1109/TMI.2019.2962361 | Google Scholar 31880546

[B3] Clowsley A. H. Kaufhold W. T. Lutz T. Meletiou A. Di Michele L. Soeller C. (2020). Detecting Nanoscale Distribution of Protein Pairs by Proximity-dependent Super-resolution Microscopy. J. Am. Chem. Soc. 142, 12069–12078. 10.1021/jacs.9b03418 PubMed Abstract | 10.1021/jacs.9b03418 | Google Scholar 32551615

[B4] Falk T. Mai D. Bensch R. Çiçek Ö. Abdulkadir A. Marrakchi Y. (2019). U-net: Deep Learning for Cell Counting, Detection, and Morphometry. Nat. Methods 16, 67–70. 10.1038/s41592-018-0261-2 PubMed Abstract | 10.1038/s41592-018-0261-2 | Google Scholar 30559429

[B5] Guo S.-M. Veneziano R. Gordonov S. Li L. Danielson E. Perez de Arce K. (2019). Multiplexed and High-Throughput Neuronal Fluorescence Imaging with Diffusible Probes. Nat. Commun. 10, 4377. 10.1038/s41467-019-12372-6 PubMed Abstract | 10.1038/s41467-019-12372-6 | Google Scholar 31558769 PMC6763432

[B6] Huang K. Demirci F. Batish M. Treible W. Meyers B. C. Caplan J. L. (2020). Quantitative, Super-resolution Localization of Small RNAs with sRNA-PAINT. Nucleic Acids Res. 48, e96. 10.1093/nar/gkaa623 PubMed Abstract | 10.1093/nar/gkaa623 | Google Scholar 32716042 PMC7498346

[B7] Jin L. Liu B. Zhao F. Hahn S. Dong B. Song R. (20201934). Link to External Site, This Link Will Open in a New Window. Nat. Commun. 11. Deep learning enables structured illumination microscopy with low light levels and enhanced speed. 10.1038/s41467-020-15784-x 10.1038/s41467-020-15784-x | Google Scholar

[B8] Liu N. Dai M. Saka S. K. Yin P. (2019). Super-resolution Labelling with Action-PAINT. Nat. Chem. 11, 1001–1008. 10.1038/s41557-019-0325-7 PubMed Abstract | 10.1038/s41557-019-0325-7 | Google Scholar 31527848 PMC6815262

[B9] Nehme E. Weiss L. E. Michaeli T. Shechtman Y. (2018). Deep-STORM: Super-resolution Single-Molecule Microscopy by Deep Learning. Optica 5, 458–464. 10.1364/OPTICA.5.000458 10.1364/OPTICA.5.000458 | Google Scholar

[B10] Ouyang W. Aristov A. Lelek M. Hao X. Zimmer C. (2018). Deep Learning Massively Accelerates Super-resolution Localization Microscopy. Nat. Biotechnol. 36, 460–468. 10.1038/nbt.4106 PubMed Abstract | 10.1038/nbt.4106 | Google Scholar 29658943

[B11] Pau G. Fuchs F. Sklyar O. Boutros M. Huber W. (2010). EBImage--an R Package for Image Processing with Applications to Cellular Phenotypes. Bioinformatics 26, 979–981. 10.1093/bioinformatics/btq046 PubMed Abstract | 10.1093/bioinformatics/btq046 | Google Scholar 20338898 PMC2844988

[B12] Ronneberger O. Fischer P. Brox T. (2015). “U-net: Convolutional Networks for Biomedical Image Segmentation,” in Medical Image Computing and Computer-Assisted Intervention. Editors Navab N. Hornegger J. Wells W. M. Frangi A. F. (Springer International Publishing), 234–241. 10.1007/978-3-319-24574-4_28 10.1007/978-3-319-24574-4_28 | Google Scholar

[B13] Schnitzbauer J. Strauss M. T. Schlichthaerle T. Schueder F. Jungmann R. (2017). Super-resolution Microscopy with DNA-PAINT. Nat. Protoc. 12, 1198–1228. 10.1038/nprot.2017.024 PubMed Abstract | 10.1038/nprot.2017.024 | Google Scholar 28518172

[B14] Wang H. Rivenson Y. Jin Y. Wei Z. Gao R. Günaydın H. (2019). Deep Learning Enables Cross-Modality Super-resolution in Fluorescence Microscopy. Nat. Methods 16, 103–110. 10.1038/s41592-018-0239-0 PubMed Abstract | 10.1038/s41592-018-0239-0 | Google Scholar 30559434 PMC7276094

[B15] Weigert M. Schmidt U. Boothe T. Müller A. Dibrov A. Jain A. (2018). Content-aware Image Restoration: Pushing the Limits of Fluorescence Microscopy. Nat. Methods 15, 1090–1097. 10.1038/s41592-018-0216-7 PubMed Abstract | 10.1038/s41592-018-0216-7 | Google Scholar 30478326

[B16] Yan Y. Liu Y. Wu Y. Zhang H. Zhang Y. Meng L. (2022). Accurate Segmentation of Breast Tumors Using AE U-Net with HDC Model in Ultrasound Images. Biomed. Signal Process. Control. 72, 103299. 10.1016/j.bspc.2021.103299 10.1016/j.bspc.2021.103299 | Google Scholar

